# Long-term influence of recurrent acute otitis media on neural involuntary attention switching in 2-year-old children

**DOI:** 10.1186/s12993-015-0086-4

**Published:** 2016-01-04

**Authors:** Sini Haapala, Elina Niemitalo-Haapola, Antti Raappana, Tiia Kujala, Kalervo Suominen, Eira Jansson-Verkasalo, Teija Kujala

**Affiliations:** 1 Department of Speech and Language Pathology, Faculty of Social Sciences, Publicum, University of Turku, 20014 Turku, Finland; 2 Clinical Neurophysiology, Oulu University Hospital, PO Box 21, 90029 Oulu, Finland; 3 Child Language Research Center and Logopedics, Faculty of Humanities, University of Oulu, PO Box 1000, 90014 Oulu, Finland; 4 Department of Otolaryngology–Head and Neck Surgery, Oulu University Hospital, PO Box 21, 90029 Oulu, Finland; 5 Department of Otolaryngology, University of Oulu, PO Box 5000, 90014 Oulu, Finland; 6 Cicero Learning, University of Helsinki, PO Box 9, 00014 Helsinki, Finland; 7 Cognitive Brain Research Unit, Institute of Behavioural Sciences, University of Helsinki, PO Box 9, 00014 Helsinki, Finland

**Keywords:** Otitis media, Involuntary attention, Orienting, ERPs, P3a, Late negativity

## Abstract

**Background:**

A large group of young children are exposed to repetitive middle ear infections but the effects of the fluctuating hearing sensations on immature central auditory system are not fully understood. The present study investigated the consequences of early childhood recurrent acute otitis media (RAOM) on involuntary auditory attention switching.

**Methods:**

By utilizing auditory event-related potentials, neural mechanisms of involuntary attention were studied in 22–26 month-old children (N = 18) who had had an early childhood RAOM and healthy controls (N = 19). The earlier and later phase of the P3a (eP3a and lP3a) and the late negativity (LN) were measured for embedded novel sounds in the passive multi-feature paradigm with repeating standard and deviant syllable stimuli. The children with RAOM had tympanostomy tubes inserted and all the children in both study groups had to have clinically healthy ears at the time of the measurement assessed by an otolaryngologist.

**Results:**

The results showed that lP3a amplitude diminished less from frontal to central and parietal areas in the children with RAOM than the controls. This might reflect an immature control of involuntary attention switch. Furthermore, the LN latency was longer in children with RAOM than in the controls, which suggests delayed reorientation of attention in RAOM.

**Conclusions:**

The lP3a and LN responses are affected in toddlers who have had a RAOM even when their ears are healthy. This suggests detrimental long-term effects of RAOM on the neural mechanisms of involuntary attention.

## Background

About 30 % of children have recurrent middle ear infections (recurrent acute otitis media, RAOM) in their early childhood [1, 2]. Due to challenges in diagnosing and classifying middle ear status, otitis media (OM) is commonly used as a general term for various forms of middle ear fluid and inflammation. A general definition for RAOM has been three or more episodes of acute otitis media (AOM) per 6 months or four or more AOM episodes per year [3]. After an episode of AOM, middle ear fluid is present for few days to over 2 months [4]. Fluid in the middle ear causes about 20–30 dB conductive hearing loss [5], and, especially when asymmetric, it affects interaural temporal and level difference cues compromising binaural sound localization [6]. Fluctuating hearing sensations during the development of central auditory system has been connected to atypical auditory processing [7–11], which can lead to problems in language acquisition [12]. Therefore, it is necessary to get a better understanding of the consequences of early childhood RAOM on immature central nervous system.

Behavioral studies in children with OM have shown problems in regulation of auditory attention [13–18]. Involuntary orientation to environmental events as well as selective maintenance of attention is essential for speech processing and language learning. Involuntary attention accounts for the detection and selection of potentially biologically meaningful information of events unrelated to the ongoing task [19]. For example, a screeching noise of a braking car causes an attention switch of a pedestrian who is talking on the phone, and leads to the distraction of the ongoing activity. After the evaluation of the irrelevant novel stimulus, the reorientation back to the recent activity takes place. Involuntary attention is a bottom-up (stimulus-driven) process [19] but during maturation the developing top-down mechanisms start to inhibit distractors which are not meaningful, in other words, children learn to separate relevant from irrelevant stimuli [20, 21]. An excessive tendency to orient to the irrelevant stimuli requiring a lot of attentional resources makes goal-directed behavior harder [22].

School-aged children with OM history were shown to have deficits of selective auditory attention in dichotic listening tasks [13–15]. They also showed increased reorientation time of attention during behavioral tasks [16]. Rated by their teachers, school-children with OM history were suggested to be less task-oriented [17] but not in all studies [23]. Studies in toddlers are scarce, probably due to the weak co-operation skills in children at this age. However, toddlers with chronic OM were shown to express reduced attention during book reading at the time of middle ear effusion and, according the questionnaire, their mothers rated them as easily distractible [18]. The neural mechanisms beyond these findings are still unknown.

Event-related potentials (ERPs) are a feasible approach for studying non-invasively neural mechanisms of involuntary auditory attention without tasks requiring co-operation skills [24]. The auditory P3a is a large positive deflection elicited by unexpected, novel sounds which substantially differ from other sounds, for example slam of the door or human cough. The P3a reflects involuntary attention mechanisms and orientation of attention [22]. It peaks fronto-centrally at 200–300 ms after the onset of a distracting stimulus [22, 25, 26].

P3a was often found to be biphasic [22, 27]. Two phases, early and late, have been identified in children [28–30] already at the age of 2 years [31]. Early P3a (eP3a) was suggested to reflect the automatic detection of violation in the neural model of existing world and thus, to represent the orientation of attention [32]. It is maximal at vertex and diminishes posteriorly and laterally [33]. In contrast, late P3a (lP3a) was suggested to reflect actual attention switch and it is maximal frontally [33]. Morphology of these responses is quite similar in children and adults but the scalp topography of children’s P3a is more anterior than that of adults [30]. The eP3a may mature earlier than lP3a, which continues to enhance frontally during development [34]. Hence, processing of acoustic novelty in the childhood resembles that in the adulthood although some underlying neural networks still continue to develop. Atypical P3a responses have been connected to abnormal involuntary attention, for example, parietally enhanced lP3a was found in children with attention deficit hyperactivity disorder (ADHD) [29].

The ERP waveform also reflects reorienting of attention back to the primary task after recognizing and evaluating a distracting stimulus [35, 36]. In adults, P3a is followed by reorienting negativity (RON) [37]. A counterpart of RON in children was suggested to be the late negativity (LN, also called as negative component, Nc) [28–31]. The LN latency, peaking at around 400–700 ms after the onset of a novel stimulus, reflects reorienting time [21]. The LN has the maximal amplitude at fronto-central scalp areas [21]. Large LN reflects enhanced neural effort to reorienting [37] or more attention paid to the surprising event [30]. During maturation, the LN amplitude has been suggested to decrease [30, 34].

The aim of this study was to compare the involuntary attentional mechanisms in 2-year-old healthy children with RAOM history and their healthy age-matched controls by recording auditory ERPs. For that purpose, novel stimuli were embedded in the multi-feature paradigm with syllables to elicit eP3a, lP3a, and LN. It was hypothesized that children with RAOM would show atypically enhanced and/or short latency P3a reflecting enhanced distractibility for the intrusive novel sounds and to have larger amplitude and/or longer latency of LN indicating more neural effort to reorienting and/or longer re-orientation time of attention than their healthy peers. Studies of P3a and novelty-related LN in toddlers are scarce and to our knowledge, this is the first study measuring these responses in children with RAOM.

## Methods

### Participants

Twenty-four children with a middle ear infection history were recruited to the RAOM group (at least three AOM per 6 months or four AOM per 1 year) from the Ear, Nose and Throat clinic of Oulu University hospital. During 1 year in 2009–2010, all children aged 22–26 months fulfilling the criteria of this study with a tympanostomy tube insertion participated (for a more detailed AOM history see [7]). The EEG recording was done on average 33 days (range 20–56 d) after the tympanostomy tube insertion. Twenty-two age matched control children with 0–2 AOM were recruited with public advertisements. All families participated voluntarily to the study and an informed written consent was obtained from the parents of children. Families were paid 15€ for travelling costs. The study was in accordance of Declaration of Helsinki and approved by the Ethics Committee of Northern Osthrobotnia Hospital District (reference number 6/2009).

Participants were from monolingual Finnish-speaking families. They were born full-term with normal birth weight, and developing typically in their sensory, cognitive, and motor skills according to parental questionnaires and the examinations at the family and health care clinics during the first 2 years of life. No family history of speech, language, or other developmental impairments or severe neuropsychiatric diseases was allowed. The standardized Finnish version of Reynell Developmental Language Scales III, the Comprehension scale [38, 39] was applied to exclude developmental language disorders. At the time of the EEG recording, transient evoked otoacoustic emissions (TEOAEs; nonlinear click sequence 1.5–4.5 kHz, 73 dB SPL, pass/refer result; MADSEN AccuScreen^®^ pro, GN Otometrics, Taastrup, Denmark) were checked. Four children with RAOM and six control children did not co-operate in the TEOAE measurement, but all the children had passed a TEOAE screening at a postnatal period in Oulu University Hospital. Right before the EEG recording, all children were assessed with pneumatic otoscopy and if needed by tympanometry and/or otomicroscopy by an otolaryngologist to ensure that they had clinically healthy ears at the time of the measurement.

In the RAOM group, one child was excluded because of a family history of dyslexia and one because the results of the Reynell III did not meet the criteria for normal speech comprehension, and an additional examination of speech-language pathologist showed signs of severe language disorder. Two children with RAOM had atypically enhanced P3a responses (24.07 and 23.20 µV), and the statistical analysis indicated them to be outliers, i.e., their responses being at abnormal distance from the other ones (2.51–17.63 µV). Because we hypothesized that the children with RAOM would have enhanced P3a responses, we decided to exclude these two children from the further analysis to avoid the bias of these extreme values on the results of the RAOM group. Furthermore, two children did not arrive to the measurement at the appointed time. In the control group, two children had to be excluded from the analysis because of a large amount of alpha activity in their EEG leading to low signal-to-noise ratio. One control children was excluded because of acute OM diagnosed at the time of measurement. The total number of children in this study was 18 in the RAOM group and 19 in the control group after these exclusions. There were no significant differences between the final groups in gender (RAOM: 10 boys; controls 11 boys), age (RAOM: mean 24 months, min–max 22–26; controls: mean 24 months, min–max 22–26), or mother’s education (RAOM: 4 low, 13 middle or high; controls 2 low, 17 middle or high). The educational information of one mother in the RAOM group was not available.

### Stimuli and experimental design

ERPs were recorded in a passive condition with the multi-feature paradigm (“Optimum-1”), which was shown to be a fast and eligible method for obtaining several ERPs reflecting different stages of auditory processing in adults [40–42], school-aged children [43, 44], and toddlers [45, 46]. In the multi-feature paradigm, the standard and the deviant sounds are presented in the same sequence so that every other stimulus is the standard and every other stimulus is one of the several deviants. In the deviants, only one sound feature (e.g., vowel or frequency) of the standard stimulus is changed at a time while the other features remain the same and strengthen the memory representation of the standard stimulus. To study attentional mechanisms, distracting novel sounds may also be embedded in the same sound stream [42, 45, 46].

The standards were Finnish semisynthetic consonant–vowel syllables/ke:/or/pi:/(duration 170 ms). Every other stimulus sequence included standard/ke:/and every other included standard/pi:/. The deviants (duration 170 ms) were five different deviations in these syllables (frequency F0 ± 8 Hz, intensity ± 7 dB, consonant from/ke:/to/pe:/and from/pi:/to/ki:/, vowel from/ke:/to/ki:/and from/pi:/to/pe:/, and vowel duration from syllable length of 170 ms to 120 ms) [42, 47]. The obligatory and MMN responses elicited by standards and deviants were reported earlier [7]. In addition, there were totally differing novel sounds (duration 200 ms, including a fall and a rise time of 10 ms), which were non-synthetic, environmental human (e.g. coughs and laughs) or non-human (e.g. door slamming and telephone ringing) sounds [42]. In the stimulus sequence, every other stimulus was a standard (probability 50 %) and every other was one of the deviants (probability 8.3 % for each) or a novel sound (probability 8.3 %). The presentation of stimuli was pseudo-randomized so that all five deviants and one novel stimulus appeared once among 12 successive stimuli, and the same deviant or novel was never repeated after the standard stimulus following it. The stimulus onset asynchrony was 670 ms. The stimuli were in the sequences lasting for about 6 min., each starting with 10 standards, and including 540 stimuli from which 275 were standards and 44 were novels sounds, the rest being deviant syllables (44 of each deviant type). Three to four stimulus sequences were presented to each participant.

Stimuli were presented in an electrically shielded and sound-attenuated room (reverberation time .3 s, background noise level 43 dB) with the sound pressure level of 75 dB via two loudspeakers (Genelec^®^ 6010A, Genelec Ltd., Iisalmi, Finland). The loudspeakers were in front of the child at a distance of 1.3 m and in a 40-degree angle from the child’s head.

### EEG recording

The EEG (.16–1000 Hz, sampling rate 5000 Hz) was recorded with 32 channel electro-cap with Ag–AgCl electrodes placed according to the international 10/20 system (ActiCAP 002 and Brain Vision BrainAmp system and software; Brain Products GmbH, Gilching, Germany). The FCz electrode served as online reference and impedances were kept below 20 kΩ. Additional electrodes placed above the outer canthus of the right eye and below the outer canthus of the left eye with bipolar montage were used for electro-oculogram.

Toddlers sat in a chair or in their parent’s lap, watching voiceless cartoons or children’s books, or played with silent toys. The parents were instructed to be as quiet as possible. The recording was camera monitored from the next room and an experienced EEG technician monitored the quality of the EEG signal during recording. During the same recording session, the children participated in an EEG recording with three to four stimulus sequences with background noise [48]. The total examination time for each participant was about two and half hours, from which the EEG registration took about 45 min. There were breaks with refreshments between the stimulus sequences.

### Analysis

Brain Vision Analyzer 2.0 (BrainProducts, GmpH) was used for offline analysis. Data were down sampled to 250 Hz and re-referenced to the average of the mastoid electrodes. Band pass filtering of .5–45 Hz, 24 dB/oct was applied to avoid aliasing and signals not originated from the brain [49]. After visual inspection, channels Fp1, Fp2, PO9, PO10, O1, Oz, and O2 were disabled from further analyzis because of artefacts. Ocular correction was done with an independent component analysis. Extracerebral artefacts with voltage exceeding ±150 μV at any electrode were removed and data were filtered with band pass of 1–20 Hz, 48 dB/oct. ERPs for standard and novel stimuli were averaged from baseline corrected EEG epochs of –100 ms prestimulus to 670 ms after stimulus onset. The first 10 standard stimuli in each recorded sequence and the standard stimuli right after the novel stimuli were excluded from the analysis. Two-tailed *t*-test indicated no significant group differences in the mean number of averaged epochs for standards or novels. The mean number of epochs for standard and novel stimuli in the RAOM group was 675 (min–max 373–856) and 133 (min–max 75–170), respectively, and in the control group 719 (min–max 517–856) and 143 (min–max 99–171), respectively.

To identify the P3a and LN, ERPs for standards were subtracted from those for novels. The grand average difference waves showed the biphasic P3a elicited by novel stimuli. Hence, eP3a and lP3a were separately analyzed. The channel selection for the peak detection was done after visual inspection, which showed the most prominent eP3a at the Cz electrode and lP3a and LN at the Fz electrode. The peak detection was done individually for each child within time windows of 180–300 ms for eP3a, 300–440 ms for lP3a, and 420–600 ms for LN. The peak latencies were determined from the most positive (eP3a and lP3a) or the most negative (LN) peak within those windows, and the mean peak amplitudes were calculated from ±20 ms time window around the peak latencies.

The statistical analyses were done for the F3, Fz, F4, C3, Cz, C4, P3, Pz, and P4 electrodes. The existence of each ERP was determined by comparing its amplitude to zero with a two-tailed *t*-test. The amplitude differences between the groups were examined with repeated measures analysis of variance (ANOVA) and the Fisher’s least significant difference (LSD) post hoc test. In ANOVA, between-subject factor was group (RAOM vs. control) and within-subject factors were anterior-posterior (AP; F3-Fz-F4 vs. C3-Cz-C4 vs. P3-Pz-P4) and right-left (RL; F3-C3-P3 vs. Fz-Cz-Pz vs. F4-C4-P4) electrode positions. The Huynh–Feldt correction was applied when appropriate. One-way ANOVA was used for studying latency differences between the groups. For the effect-size estimation, the partial eta squared (*ƞ*_*p*_^*2*^) was calculated.

## Results

**Fig. 1:**
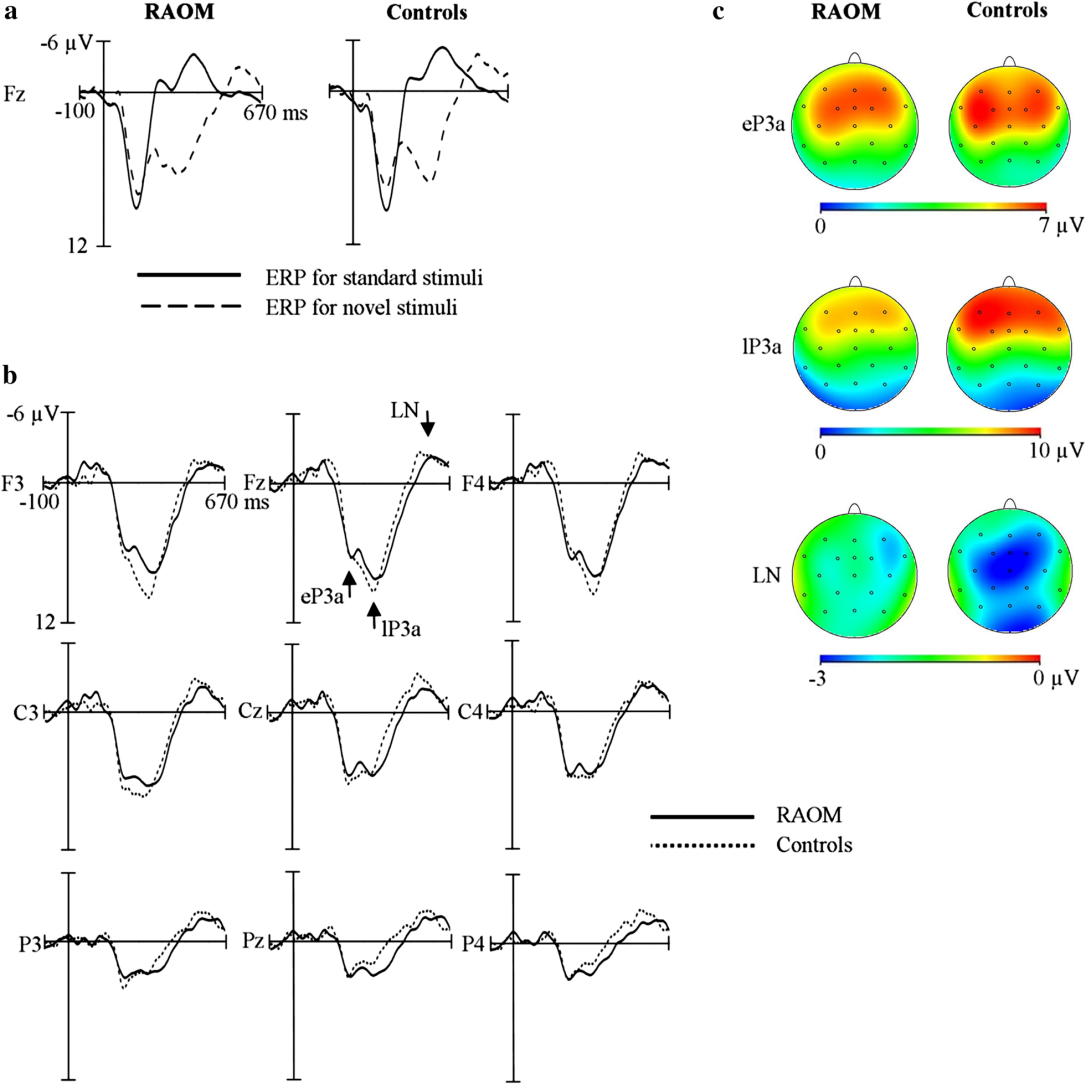
ERPs (eP3a, lP3a, and LN) elicited by novel stimuli in children with recurrent acute otitis media (RAOM) and their controls; **a** grand average standard and novel ERP waves, **b** grand average difference (novel minus standard ERP) waves, and **c** scalp topographies

The eP3a significantly differed from zero in the children with RAOM and in the controls (two tailed *t*-test; *p* ≤ .001; Table 1, Fig. 1) with no group differences in the amplitude, amplitude scalp distribution, or latency. In both groups, the eP3a amplitude was stronger at the frontal and central electrodes than at the parietal electrodes (*F* [2, 57] = 42.09, *p* < .001, *ƞ*_*p*_^*2*^ = .53; LSD post hoc *p* < .001).

**Table 1:** Mean amplitude and latency of ERPs elicited by novel stimuli in children in both groups

	Electrode	Amplitude µV		Latency ms	
		RAOM	Control	RAOM	Control
eP3a	Cz	6.66 (4.30)	6.97 (3.44)	244 (26)	248 (25)
lP3a	Fz	9.30 (3.69)	9.67 (3.74)	348 (36)	341 (22)
LN	Fz	–2.82 (3.38)	–2.34 (3.04)	*599* (40)	*526* (43)

Standard deviations are in parentheses

A significant group difference is italized

*RAOM* recurrent acute otitis media

There was a significant lP3a in both groups (two tailed *t*-test; *p* ≤ .001; Table 1, Fig. 1). A repeated measures ANOVA for the lP3a amplitude indicated a significant AP x group interaction (*F* [2, 59] = 3.94, *p* = .03, *ƞ*_*p*_^*2*^ = .10). According to the LSD post hoc test, the children with RAOM showed a more even AP distribution than the control children who had a clear frontally maximal and posteriorly diminishing amplitude scalp distribution (mean amplitudes frontally 8.99 vs. 9.67 µV, centrally 7.12 vs. 6.04 µV, and parietally 3.64 vs. 2.17 µV in the RAOM and control groups, respectively). Furthermore, a significant AP x RL interaction with no group difference was found (*F* [4, 140] = 4.98, *p* < .001, *ƞ*_*p*_^*2*^ = .13). LSD post hoc test indicated an even RL amplitude distribution at frontal electrodes, but stronger left hemispheric activation compared to the vertex and the right line at central electrodes (*p* < .001) and also stronger left than right line responses at parietal electrodes (*p* = .03). There was no group difference for the lP3a latency.

In both groups, a significant LN was found (two tailed *t*-test; RAOM: *p* = .001; control: *p* = .004; Table 1, Fig. 1). A repeated measures ANOVA for the LN amplitude indicated a significant AP × RL interaction (*F* [4, 148] = 2.96, *p* = .02). This was due to the weakest amplitude at F3 (LSD post hoc; *p* = .001–.03) and the strongest amplitude at Cz (LSD post hoc; *p* = .001–.002). There were no group differences in the amplitude or amplitude scalp distribution of LN. However, one-way ANOVA indicated a significant group difference in the LN latency (*F* [1, 37] = 32.76, *p* < .001, *ƞ*_*p*_^*2*^ = .47), which peaked later in the children with RAOM than in the controls.

## Discussion

This study examined the effects of early childhood RAOM on neural mechanisms of involuntary attention at the age of 2 years. For that purpose, the P3a and LN elicited by distracting novel sounds were measured in the linguistic multi-feature paradigm at the time when all the participants had healthy ears and their sound encoding reflected by obligatory ERPs was found to be intact in an earlier study [7]. Both the children with RAOM and the controls showed a clearly identifiable P3a with two phases (eP3a and lP3a) and a LN, the morphology of which was found to be consistent with earlier studies in children [28–31, 45, 46]. However, the topography and timing of these responses were distinct in the two groups. These findings suggest different maturational trajectories in the two groups of children and suggest that the consequences of OM are not limited to the middle ear effusion period but the effects are long-lasting.

The amplitude, distribution, or latency of the eP3a did not differ between the groups. This suggests the similar automatic detection of a novel stimulus and the early stages of the orientation of attention [32] in the groups. The eP3a was larger frontally and centrally than parietally in both groups being in line with earlier studies in typically developed school-aged children [28, 34] and in adults [33].

In contrast, a significant group difference was found in the lP3a reflecting the actual attention switch [33]. The lP3a amplitude diminished less in the children with RAOM than in the controls from frontal to central and parietal areas, which may indicate an immature control of attention switch in children with RAOM. A frontally prominent lP3a has been linked to the neural maturation of the frontal cortex and attention control [28, 34]. Likewise, an enhanced lP3a at the posterior scalp areas has earlier been found in easily distractible children with ADHD [29]. The current result supports the behavioural finding on the distractibility of toddlers with OM [18]. Distractibility can lead to weak utilization of the auditory channel in learning [29, 50], since it limits the ability to ignore irrelevant auditory stimuli. At 2 years of age, this may contribute to the emerging language by disrupting child’s engagement with social-communicative actions critical for language learning.

The LN latency was longer in the children with RAOM than in the controls suggesting delayed reorienting back to the ongoing activity [30, 34]. This corresponds with previous results suggesting delayed reorienting in school-children in a behavioral test [16] and might indicate that children with RAOM have an abnormally low resistance to auditory distraction. This is supported by our previous results suggesting neural sensitivity to sound loudness changes in these same 2-year-old children with RAOM [7]. The result is also consistent with the elevated auditory sensitivity to sounds described in adolescents with childhood OM [51].

Our results show that RAOM has long-term effects leading to abnormal attention control at the age of 2 years when rapid developmental neural changes are involved. Studies on attentional neural mechanisms in older children with early childhood RAOM would be pertinent since they would disclose whether the neural changes observed are transient or still persisting at the later stages of development.

There were two children in the RAOM group who were excluded from the group analysis because of their abnormally enhanced P3a responses. The exclusion was done to avoid the bias of these statistically confirmed outliers on the results of the RAOM group. However, we should notice that these extreme P3a responses might reflect a genuine effect of RAOM and indicate enhanced distractibility of these children, but this should be studied further in the future.

When interpreting the results, it should be taken into account that the accurate hearing thresholds were not available at the time of the measurement. Accurate hearing thresholds can be reliably measured from the age of three onwards [52]. Because the participants in the current study were 22–26 months old we decided to use TEOAE screening to exclude congenital hearing losses. However, there were six children in the RAOM group and four children in the control group who could not tolerate the TEOAE measurement at the time of EEG. Because these children had passed the TEOAE screening at the postnatal period, we decided to include them in the study. However, there is a possibility that a child who has passed the TEOAE screening at birth may develop hearing deficit later. Hearing levels were assumed to be at normal levels in all participants while the children with RAOM had had tympanostomy tubes inserted and, according the parental reports, there were no concerns of hearing in the screenings at the family and health care clinics where Finnish children are followed up regularly. Furthermore, these same children showed age-typical cortical sound encoding with no group differences in our earlier study [7]. This refers to hearing levels within the normal range at the time of the EEG.

## Conclusions

To conclude, this study showed abnormal neural mechanisms of involuntary attention in 2-year-old children with RAOM. For the distracting novel sounds, the RAOM group showed atypical neural organization signified by a more even lP3a scalp distribution in anterior-posterior axis than in the controls, who had a more frontally oriented lP3a. This can be a sign of immature neural processing and enhanced distractibility. Furthermore, the children with RAOM showed delayed re-orienting back to the ongoing activity indicated by their prolonged LN latency. Since all the children had clinically healthy ears at the time of the study, the current results suggest that early childhood RAOM has long-term effects on the immature central nervous system. This further supports the suggestion that early childhood RAOM should be taken as a risk factor for the developing auditory central nervous system.

## Abbreviations

LN: late negativity
OM: otitis media
RAOM: recurrent acute otitis media
